# VDAC1 Inhibition Protects Against Noise‐Induced Hearing Loss via the PINK1/Parkin Pathway

**DOI:** 10.1111/cns.70410

**Published:** 2025-04-26

**Authors:** Yuchen Jin, Wenqi Dong, Yumeng Jiang, Lingkang Dong, Zhuangzhuang Li, Dongzhen Yu

**Affiliations:** ^1^ Shanghai Key Laboratory of Sleep Disordered Breathing, Department of Otolaryngology‐Head and Neck Surgery, Otolaryngology Institute of Shanghai Jiao Tong University Shanghai Sixth People's Hospital Affiliated to Shanghai Jiao Tong University School of Medicine Shanghai China; ^2^ Department of Otolaryngology, Sun Yat‐Sen Memorial Hospital Sun Yat‐Sen University Guangzhou China

**Keywords:** 4,4′‐Diisothiocyanostilbene‐2,2′‐disulfonic acid, autophagy, hair cell, noise‐induced hearing loss, reactive oxygen species

## Abstract

**Aims:**

This study examined the effect of 4,4′‐diisothiocyanostilbene‐2,2′‐disulfonic acid (DIDS), an anion channel blocker of voltage‐dependent anion channel 1 (VDAC1), on noise‐induced hearing loss (NIHL) and its underlying mechanisms.

**Methods:**

Cochlear explants and House Ear Institute‐Organ of Corti 1 (HEI‐OC1) cells were used to assess the effect of DIDS in vitro. Auditory brainstem responses were used to assess auditory functions in mice. Immunofluorescence staining of myosin 7a and CTBP2 were used to examine hair cells and synaptic ribbons. The accumulation of reactive oxygen species (ROS) was measured by 4‐HNE staining. The gene expression changes of cochlea were analyzed using RNA sequencing.

**Results:**

DIDS reduced the levels of ROS in cochlear explants and attenuated cell death caused by hydrogen peroxide in both cochlear explants and HEI‐OC1 cells. In C57BL/6 mice, DIDS reduced ROS generation and tumor necrosis factor‐α induced by noise exposure, thereby protecting outer hair cells and inner hair cell synaptic ribbons from noise‐induced damage through a mechanism involving the PINK1/Parkin signaling pathway. The preventive effect of DIDS in cochlear explants was eliminated by mitophagy inhibition.

**Conclusion:**

VDAC1 inhibition enhances mitophagy in cochlear hair cells, playing a critical role in defending against oxidative stress and inflammation. Downregulation of VDAC1 may thus be considered a therapeutic strategy for preventing cochlear hair cell damage and reducing NIHL.

## Graphical abstract

VDAC1 is expressed in cochlear hair cells and its expression is increased after noise exposure. Inhibiting VDAC1 by DIDS protects cochlear hair cells and synaptic ribbons from noise‐induced damage in C57BL/6 mice. VDAC1 inhibition reduces ROS levels and inflammation in cochlear hair cells after noise. VDAC1 inhibition enhances mitophagy in cochlear hair cells via the PINK1/Parkin signaling pathway.

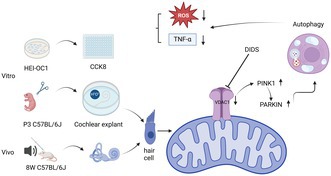

## 1 Introduction

The inner ear is an exceedingly complex yet essential component of the auditory and vestibular systems, which allows both the identification of sound and the maintenance of balance [1]. It consists of several intricate structures, each with specific functions that contribute to the overall sensory experience. Hearing loss is one of the most prevalent health conditions, affecting more than 20% of the global population. Sensorineural hearing loss (SNHL) results from damage to the auditory nerve or hair cells in the cochlea and is a common form of hearing impairment linked to genetic factors, chronic exposure to loud noises, aging, infections, and ototoxic drugs [2, 3, 4, 5, 6]. While gene therapy, cell therapy, and pharmacotherapy appear to be promising approaches for treating SNHL [7, 8, 9], its management primarily relies on hearing aids and cochlear implants. Noise‐induced hearing loss (NIHL), mainly caused by occupational noise exposure, accounts for 16% of globally disabling hearing loss [10, 11]. Prolonged exposure to high noise levels damages the delicate structures within the inner ear, including hair cells (HCs) and cochlear nerve terminals, and the synapses that connect them [12]. Noise levels exceeding 85 dB are considered harmful to the auditory organs, although individual tolerance to noise varies [13]. Depending on the intensity and duration of exposure, NIHL may manifest as either temporary or permanent threshold shifts [14]. In cases of permanent threshold shifts, noise exposure induces the programmed cell death of HCs, leading to irreversible hearing loss.

Mitochondria are integral to the respiratory and energy metabolism processes in eukaryotic cells. Additionally, they play a major role in generating reactive oxygen species (ROS) [15]. The cellular ability to maintain a healthy mitochondrial population and prevent the accumulation of damaged organelles is critical for preserving auditory function. Oxidative stress resulting from noise exposure affects the structural and functional properties of mitochondria by reducing membrane potential and adenosine triphosphate (ATP) production [16]. Mitophagy, a form of selective autophagy, is responsible for eliminating damaged or dysfunctional mitochondria and provides some degree of protection to cochlear HCs against aminoglycoside‐induced damage [17]. Furthermore, restoration of impaired mitophagy processes can facilitate HC recovery [17]. The inhibition of proteins that promote mitophagy, including PINK1/Parkin, exacerbates ototoxicity; this effect is reversed by the administration of mitophagy inducers [18]. Impaired mitophagy in the central auditory system has been implicated in age‐related progressive hearing loss [18].

Voltage‐dependent anion channel 1 (VDAC1) consists of 19 transmembrane β‐strands interconnected by flexible loops, forming a barrel‐shaped protein. The N‐terminal portion is located within the pore but can translocate to the cytoplasm [19]. Recent research suggests that VDAC1 contributes to mitochondrial damage and mitophagy in neurodegenerative and ophthalmic diseases [20, 21, 22]. However, its functional role in NIHL and the underlying pathogenic mechanisms have not been explored. Therefore, in the present study, the role of VDAC1 in NIHL and its underlying mechanisms were investigated in vitro using HEI‐OC1 cells and cochlear explants, and in vivo in adult C57BL/6 mice. A better understanding of the involvement of VDAC1 in hearing loss could lead to the development of new therapeutic strategies.

## 2 Materials and Methods

### 2.1 Animals

All animal experiments were conducted in accordance with the guidelines of the Institutional Animal Care and Use Committee of the Shanghai Sixth People's Hospital, affiliated with Shanghai Jiao Tong University School of Medicine. The experiments were performed on 3‐day‐old and 8‐week‐old C57BL/6 male mice. The mice were maintained under standard housing conditions with ambient temperatures ranging from 20°C to 24°C. Food and water were provided ad libitum.

### 2.2 Noise Exposure

Mice were exposed for 2 h to octave band noise with a frequency spectrum of 8–16 kHz at a sound pressure level (SPL) of 100 dB. During noise exposure, the mice were confined to stainless steel wire cages. Continuous monitoring of the noise level was conducted to ensure a consistent and accurate acoustic stimulus.

### 2.3 Drug Administration

4,4′‐Diisothiocyanostilbene‐2,2′‐disulfonic acid (DIDS, MedChemExpress, HY‐D0086, USA) was dissolved in dimethyl sulfoxide and stored at −20°C. Before use, it was diluted in a 0.9% saline solution. Mice were randomly assigned to one of the following three groups: (1) a control group that received an intraperitoneal (IP) injection of an equivalent volume of dimethyl sulfoxide in saline solution, (2) a group exposed to 100 dB noise (100 dB group) with an IP injection of an equivalent volume of dimethyl sulfoxide in saline solution, and (3) a group exposed to 100 dB noise and treated with DIDS (100 dB + DIDS group). Mice in the 100 dB + DIDS group received an IP injection of 14 mg DIDS/kg 2 h prior to noise exposure and immediately after noise exposure. The same dose of DIDS was administered via IP injection once daily until day 7 post‐exposure. The DIDS dose was based on previous research [23].

### 2.4 Auditory Brainstem Response (ABR)

A TDT system (Tucker Davis Technologies, USA) was used to record ABR. Eight‐week‐old C57BL/6J mice were anesthetized with an IP infusion of 1% sodium pentobarbital at a dose of 75 mg/kg body weight and then placed on a thermostatically controlled electric blanket at 37°C inside a soundproof chamber for audiometry. Subcutaneous electrodes were placed as follows: the active electrode on the head, the reference electrode at the ipsilateral ear to the stimulated ear, and the ground electrode on the hind limb. ABR tests were conducted using the SigGenRZ software from Tucker Davis Technologies across frequencies of 4, 8, 16, 22.6, and 32 kHz. The acoustic stimuli were initially set at a maximum of 90 dB SPL and subsequently reduced in 5‐dB steps down to 0 dB SPL. The ABR threshold was defined as the lowest stimulus intensity at which a wave III component could be identified in the 4–32 kHz frequency range.

### 2.5 Immunofluorescence

The organs were fixed with 4% paraformaldehyde (Sangon Biotech, A500684), permeabilized with Triton‐X 100 (Sangon Biotech, A600198), blocked for 1 h, and stained with primary antibodies targeting VDAC1 (1:250, ab15895; Abcam), myosin‐VIIa (1:500, 25‐6790; Proteus Biosciences), 4‐HNE (1:250, ab46545; Abcam), and CTBP2 (1:1000; BD Biosciences, 612044) at 4°C overnight. Samples were then incubated with appropriate secondary antibodies (Thermo Fisher Scientific, A32723, A32731, A32727, and A32732) for 1 h at room temperature in the dark. Cochlear HCs were labeled with phalloidin (1:2000, ab176753; Abcam) during a 10‐min incubation. Imaging was performed using an LSM 710 confocal microscope (Carl Zeiss, Heidelberg, Germany).

### 2.6 MitoSOX Staining

Cochlear explants were incubated with MitoSOX (Invitrogen, M36008, USA) at 37°C for 30 min in the dark.

### 2.7 Western Blotting

Proteins from mouse cochlear tissues were isolated using the Minute total protein extraction kit (Invent Biotechnologies, Eden Prairie, MN, USA) in combination with a protease inhibitor (Epizyme, GRF101, China). Protein concentrations were determined using a bicinchoninic acid protein assay kit (Epizyme, ZJ101).

Equal amounts of protein were loaded onto a 12.5% or 15% SDS‐polyacrylamide gel. The separated proteins were transferred onto BioTrace NT nitrocellulose membranes (Pall Corp., USA), blocked for 1 h at room temperature with 5% nonfat dry milk (Epizyme, PS112L) in Tris‐buffered saline containing Tween (Sangon Biotech, C006161, China), and then incubated overnight at 4°C with the following primary antibodies: VDAC1 (1:1000, ab15895; Abcam), PINK1 (1:1000, 23274‐1‐AP; Proteintech), Parkin (1:1000, 14060‐1‐AP; Proteintech), P62 (1:1000, 18420‐1‐AP; Proteintech) and LC3 (1:1000, 14600‐1‐AP; Proteintech). After a 1‐h incubation with the corresponding secondary antibodies (ABclonal, AS014, USA), bands were visualized using an imaging system (Clinx Science Instruments Co. Ltd., Shanghai, China), and the images were analyzed using ImageJ software (National Institutes of Health, Bethesda, MD, USA).

### 2.8 Hair Cell and Cochlear Ribbon Synapse Determinations

Surviving HCs were defined as those with normal nuclei and labeled with myosin‐VIIa. Cochlear HCs were imaged at 63× magnification on a Zeiss microscope, and their turns were quantified using ImageJ software. The number of ribbon synapses in the middle of the basilar membrane was determined by anti‐CTBP2 antibody staining, and CTBP2 proteins per inner HC (IHC) field were then quantified.

### 2.9 HEI‐OC1 Cell Culture

The HEI‐OC1 cell line was generously provided by Dr. Iris Heredia (Technology Development Group, UCLA). Before use, the cell line was validated by myosin‐VIIa immunohistochemistry. The cells were cultured as described in previous studies [24, 25].

### 2.10 Cell Viability

Cell viability was assessed using the Cell Counting Kit‐8 (CCK‐8, MedChemExpress, HY‐K0301) as per the manufacturer's instructions. HEI‐OC1 cells were plated in triplicate at a density of 5 × 10^3^ cells/well in 96‐well plates. Viability after H_2_O_2_ exposure was determined by replacing the culture medium with medium containing various concentrations of H_2_O_2_ (0, 0.1, 0.2, 0.5, and 1 mmol/L), followed by a 24‐h incubation. To assess the protective effects of DIDS against H_2_O_2_ toxicity, cells were incubated for 24 h in medium supplemented with different concentrations of DIDS (0, 20, 40, 100, and 200 μmol/L) along with the designated concentration of H_2_O_2_.

### 2.11 Cochlear Explants

Collagen gels were prepared by mixing collagen type 1 from rat tail (BioCoat, USA), 10× Eagle basal medium (Sigma‐Aldrich, USA), and 2% sodium carbonate at a 9:1:1 ratio. Cochleae collected from 3‐day‐old C57BL/6J mice were attached to the collagen gels, which were then placed in 2 mL of DMEM/F12 growth medium (Gibco, 11320033, USA) containing B‐27 (Gibco, 17504044, USA), N‐2 (Gibco, 17502048, USA), and ampicillin (Sangon Biotech, A610029, China) at a concentration of 50 μg/mL. After 1 day of culture at 37°C in a humidified chamber with 5% CO_2_, the explants were treated for 24 h with H_2_O_2_ with or without DIDS, as described above.

### 2.12 Immunohistochemistry Staining

The excised cochleae were fixed overnight in 4% paraformaldehyde solution at 4°C. Decalcification was performed by incubating the samples in 10% EDTA at room temperature for 24 h. After dehydration through an ethanol series, the specimens were embedded in paraffin, sectioned to a thickness of 3–5 μm, and incubated in citric acid (Servicebio, g1202, pH 6.0) for antigen retrieval. Subsequently, the tissue samples were stained with rabbit polyclonal anti‐tumor necrosis factor‐α (TNF‐α) (Servicebio, gb11188, 1:200), followed by the corresponding secondary antibody, and analyzed using a white light microscope.

### 2.13 Statistical Analysis

Statistical analyses were conducted using GraphPad Prism. Prior to conducting the analysis, the data were initially assessed for normality. The Shapiro–Wilk test was used to evaluate the distribution characteristics. The results are expressed as means ± standard deviations (SDs). Intergroup differences were assessed using a two‐tailed unpaired Student's *t*‐test for two groups and One‐Way ANOVA or Two‐Way ANOVA followed by LSD's multiple comparison test for three or more groups. The threshold for statistical significance was defined as *p* < 0.05.

## 3 Results

### 3.1 VDAC1 Expression in the Cochlear Region Is Upregulated in Response to Noise Exposure

**FIGURE 1:**
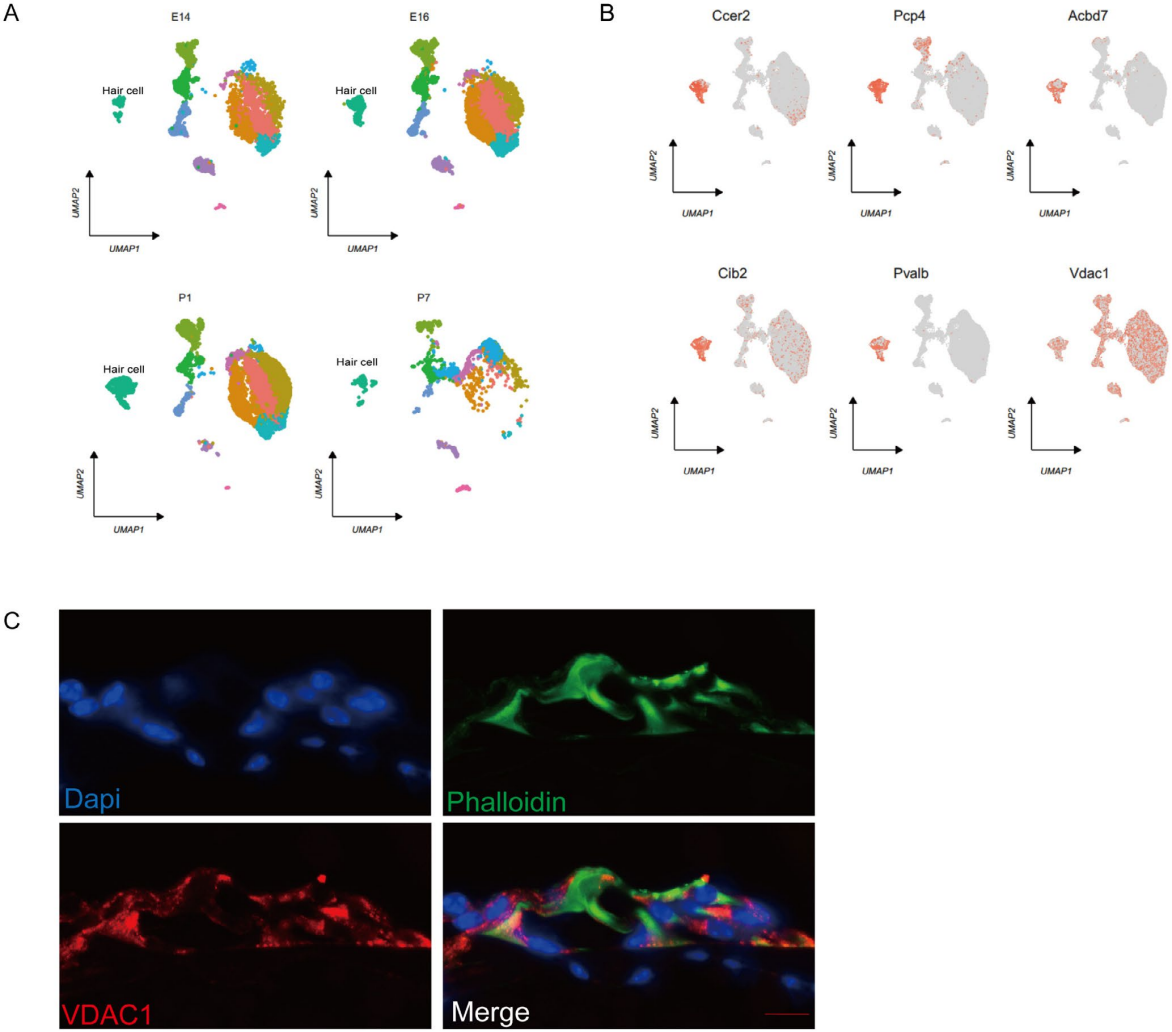
Voltage‐dependent anion channel 1 (VDAC1) expression in cochlear hair cells (HCs). (A) The Unified Manifold Approximation and Projection plot depicting four distinct time points (E14, E16, P1, P7) in the development of the mouse cochlea. (B) A feature plot showing the expression of Ccer, Pcp4, Acbd7, Cib2, Pvalb (HC markers), and VDAC1. (C) Immunofluorescence staining of frozen sections of the mouse cochlea for DAPI (blue), phalloidin (green), and VDAC1 (red) reveals high levels of VDAC1 expression in HCs. Scale bar: 10 μm.

The role of VDAC1 in HCs was investigated by first examining its expression in the HCs of C57BL/6 mice. Data from mouse cochlear samples at four specific time points (E14, E16, P1, and P7) were obtained from the GSE137299 dataset [26]. After data clustering and downsizing, annotations were assigned to the HCs using marker genes (Ccer, Pcp4, Acbd7, Cib2, and Pvalb) from recent literature [26, 27] (Figure 1A,B). Both single‐cell data and immunofluorescence analysis of frozen sections of the mouse cochlea showed that VDAC1 is widely expressed in HCs (Figure 1C).

**FIGURE 2:**
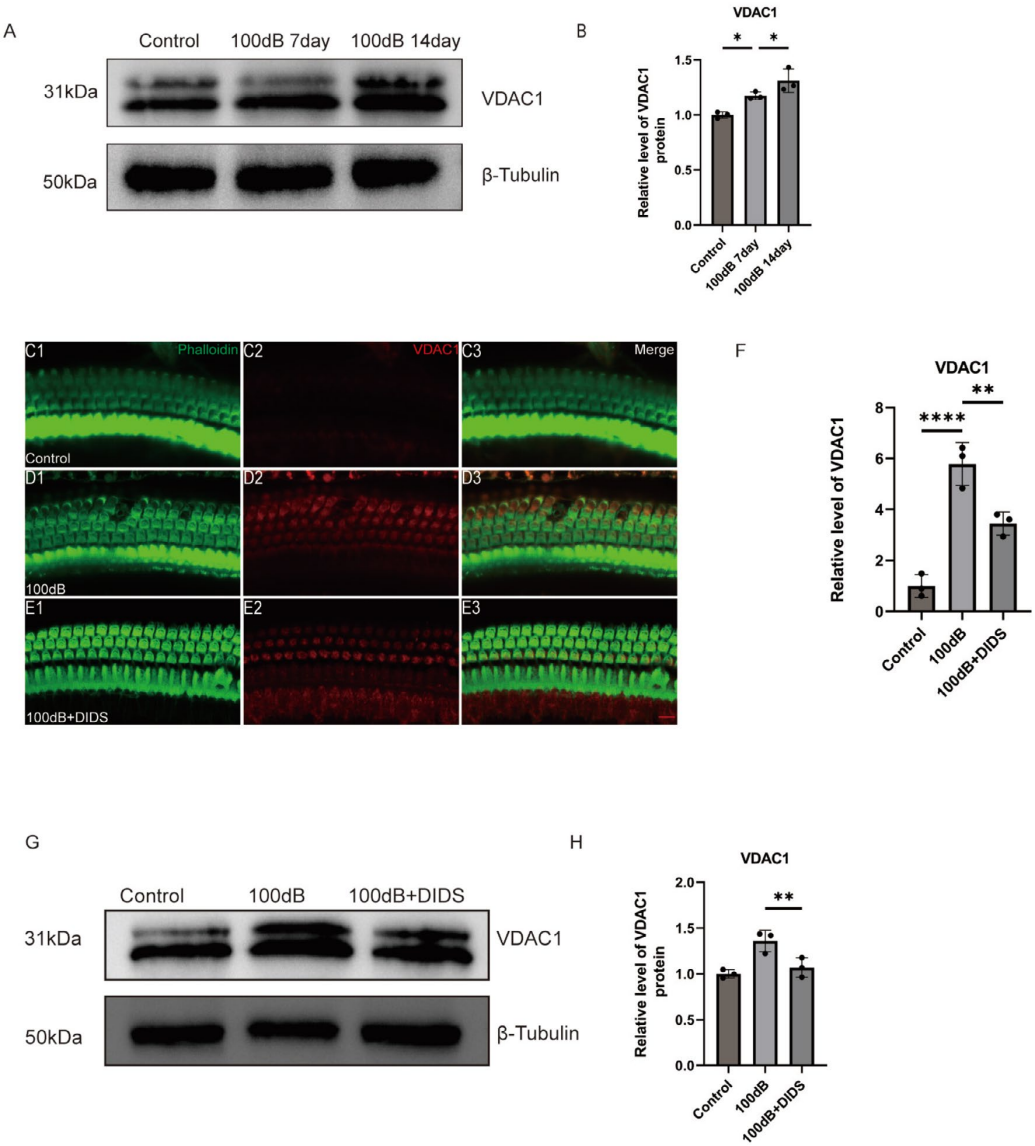
VDAC1 expression in the cochlea of C57BL/6 mice is increased by noise exposure. (A, B) Relative levels of VDAC1 determined by western blotting (*n* = 3). *p < 0.05 versus control group; *p < 0.05 versus 100 dB 7 day group. (C–E) Immunofluorescence staining for phalloidin (green) and VDAC1 (red) shows that the increase in VDAC1 expression after noise exposure is inhibited by DIDS (*n* = 3). Scale bar: 10 μm. (F) Quantitative analysis of VDAC1 expression in outer hair cells (OHCs, *n* = 3). ****p < 0.0001 versus control group; **p < 0.01 versus 100 dB group. (G, H) Relative levels of VDAC1 protein determined by western blotting (*n* = 3). **p < 0.05 versus 100 dB group.

The increased expression of VDAC1 in the cochlea of C57BL/6 mice exposed to noise from day 7 to day 14 (Figure 2A,B) demonstrates the effect of noise exposure on VDAC1 expression in vivo. DIDS, an inhibitor of anion transport, interacts with VDAC1 to prevent its oligomerization [28]. The ability of DIDS to inhibit the noise‐induced increase in VDAC1 expression was confirmed by western blot analysis and immunostaining (Figure 2C–H). Overall, these findings demonstrate an increase in VDAC1 expression in the cochlear outer hair cells (OHCs) of adult mice in response to noise exposure and its inhibition by DIDS (Figure 2C–H).

### 3.2 DIDS Mitigates the Death of HEI‐OC1 Cells and HCs Induced by H_2_O_2_

**FIGURE 3:**
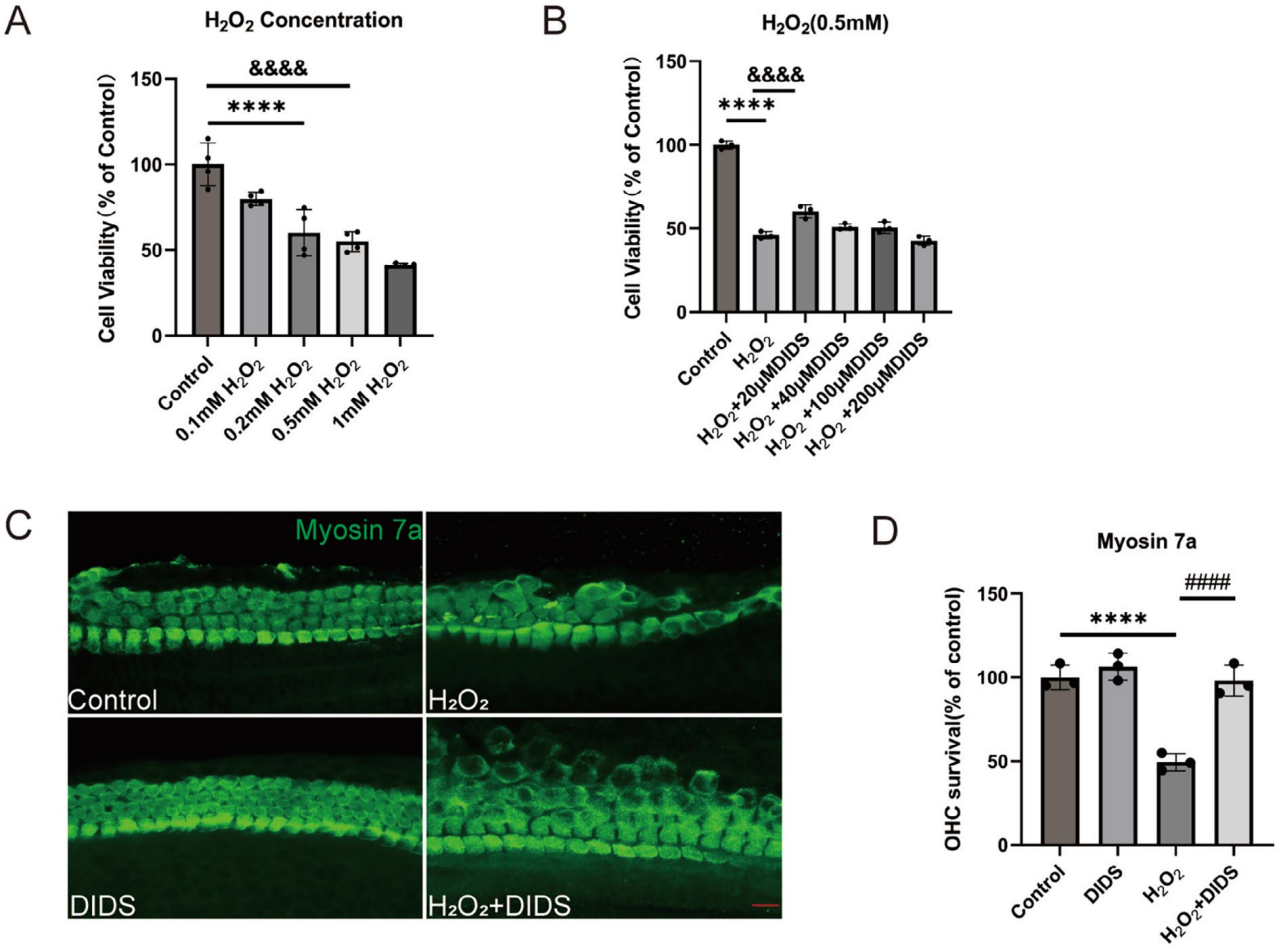
DIDS protects HEI‐OC1 cells from H_2_O_2_ damage and prevents HC loss in H_2_O_2_‐exposed cochlear explants. (A) Survival rates of HEI‐OC1 cells exposed to varying concentrations of H_2_O_2_, as determined by a CCK‐8 assay (*n* = 4). ****p < 0.0001 and ^&&&&^ *p* < 0.0001 versus control group. (B) Viability of HEI‐OC1 cells exposed to 0.5 mmol/L H_2_O_2_ and varying concentrations of DIDS, as determined by a CCK‐8 assay (*n* = 3). ****p < 0.0001 versus control group; ^&&&&^ *p* < 0.0001 versus H_2_O_2_ group. (C) Representative images of the basal turn of cochlear explants in the various experimental groups after immunolabeling with myosin‐VIIa (green). Scale bar: 10 μm. (D) Quantitative analysis of OHC survival in the different groups (*n* = 3). ****p < 0.0001 versus control group; ^####^ *p* < 0.0001 versus H_2_O_2_ group.

The efficacy of DIDS in protecting against ROS damage was investigated in vitro in experiments on cochlear explants and HEI‐OC1 cells. The viability of HEI‐OC1 cells decreased by approximately 50% following a 24‐h exposure to 0.5 mmol/L H_2_O_2_. This condition was used in all subsequent experiments because it caused cell damage while maintaining an adequate number of viable cells (Figure 3A). The protective effects of various concentrations of DIDS were then examined, revealing that a concentration of 20 μmol/L significantly mitigated the oxidative‐stress‐induced damage to HEI‐OC1 cells (Figure 3B).

The protective effects of DIDS on cochlear HCs in vitro were then assessed using cochlear explants obtained from P3 C57BL/6J mice. A previous study [29] showed a dose‐dependent negative impact of H_2_O_2_ on OHCs, but this effect was limited to the basal cochlear turn of the explants. In this study, the cultured explants were treated as follows: control (no treatment), DIDS (20 μmol/L DIDS for 24 h), H_2_O_2_ (0.5 mmol/L H_2_O_2_ for 24 h), and H_2_O_2_ + DIDS (0.5 mmol/L H_2_O_2_ and 20 μmol/L DIDS for 24 h). After treatment, myosin‐VIIa (green) staining was used to quantify the OHCs in the basal turn of the cochleae. As shown in Figure 3C, there were no significant differences in the number of OHCs between the control and DIDS groups, indicating that DIDS alone did not harm the cells. However, compared with the control group, there was a significant decrease in OHCs in the H_2_O_2_ group (Figure 3C,D). In contrast, the number of OHCs was substantially higher in the H_2_O_2_ + DIDS group than in the H_2_O_2_ group (Figure 3C,D). These findings demonstrate that DIDS protects against the damaging effects of H_2_O_2_ in HEI‐OC1 cells and prevents HC loss in cochlear explants.

### 3.3 DIDS Mitigates the Threshold Shifts in the ABR Caused by Noise Exposure by Protecting OHCs and IHC Synaptic Ribbons Against Noise‐Induced Damage

**FIGURE 4:**
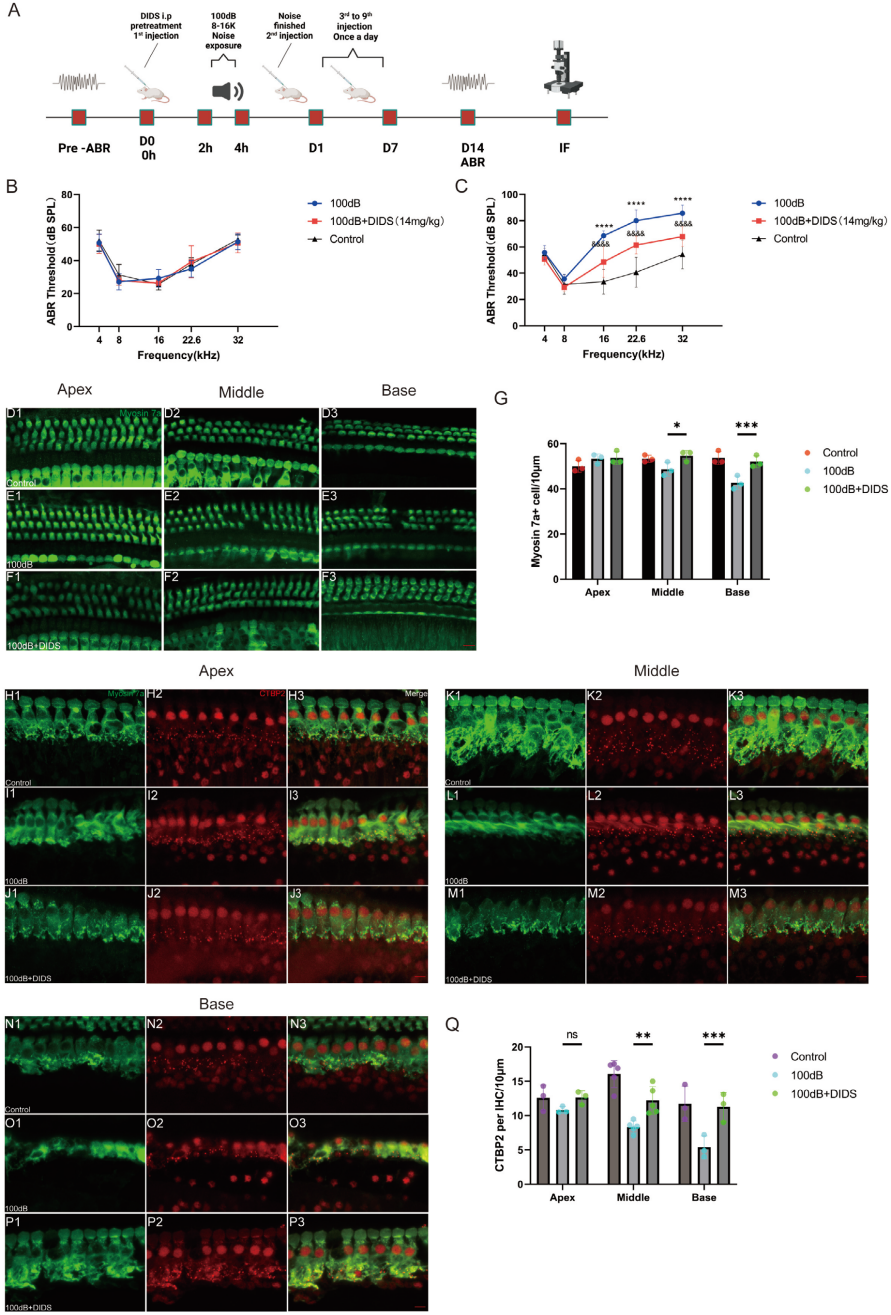
DIDS reduces noise‐induced ABR threshold shifts by protecting OHCs and IHC synaptic ribbons. (A) Experimental schedule. This figure was created with BioRender.com. (B) ABR thresholds of the different groups before treatment (*n* = 7). (C) Day 14 ABR thresholds in the different groups treated as described in (A) (*n* = 7). ****p < 0.0001 versus control group; ^&&&&^ *p* < 0.0001 versus 100 dB group. (D–F) Representative confocal images of myosin‐VIIa‐stained (green) cochlear HCs from the different groups. Scale bar: 10 μm. (G) Quantification of HCs per 100 μm in different areas of the cochlea based on myosin‐VIIa staining (*n* = 3). *p < 0.05 and ***p < 0.001 versus 100 dB group. (H–P) Representative images of immunolabeled CTBP2 in IHCs located in the three turns of the cochlea in noise‐exposed mice with or without DIDS supplementation and in the control group. Scale bar: 10 μm. (Q) Quantification of synaptic ribbons in IHCs in the middle turn (*n* = 5), apical turn (*n* = 3), basal turn (*n* = 3) of the cochlea with myosin‐VIIa (green) and CTBP2 (red). ****p < 0.001 and **p < 0.01 versus 100 dB group.

The aforementioned experiments demonstrated the protective properties of DIDS in both HEI‐OC1 cells and cochlear explants. The effect of DIDS was then tested in C57BL/6J mice, as shown in Figure 4A. Specifically, whether DIDS‐mediated inhibition of VDAC1 expression in HCs protects against NIHL was examined by measuring the ABR at various frequencies (4, 8, 16, 22.6, and 32 kHz) 14 days after noise exposure. There was no statistically significant difference in the hearing thresholds of the three groups before the experiment (Figure 4B). The results of the ABR tests are shown in Figure 4C. Hearing thresholds were significantly higher in the 100 dB group than in the control group and significantly lower in the 100 dB + DIDS group than in the 100 dB group across most of the tested frequencies (16, 22.6, 32 k).

Consistent with the ABR findings, OHCs in the middle and basal turns of the cochlea were damaged by noise exposure. However, in the 100 dB + DIDS group, a considerable decrease in HC loss in the middle and basal turns of the cochlea was observed (Figure 4D–G).

Given the well‐documented noise‐induced impairment of IHC synaptic ribbons [9], the ability of DIDS to mitigate the damage 14 days after noise exposure was examined in presynaptic ribbons immunolabeled with CTBP2. Noise exposure caused a significant loss of synaptic ribbons in the middle and basal cochlear turn compared with the 100 dB group (Figure 4H–Q), but the damage was largely attenuated by DIDS (Figure 4H–Q). These results demonstrate the ability of DIDS to reduce the ABR threshold shifts in noise‐exposed C57BL/6J mice by protecting OHCs and IHC synaptic ribbons.

### 3.4 DIDS Reduces the Oxidative Stress In Vitro and In Vivo

**FIGURE 5:**
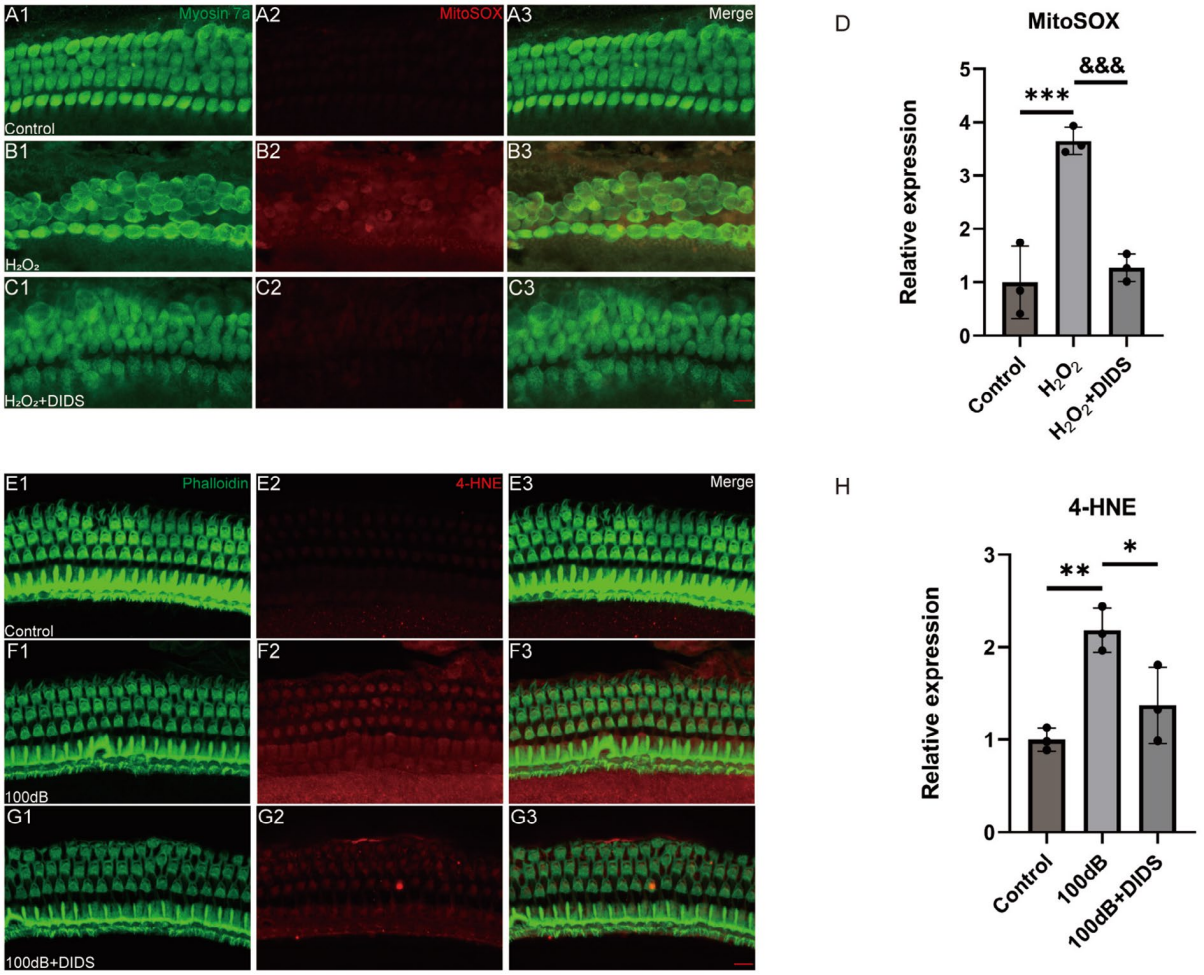
DIDS administration reduces oxidative stress in vitro and in vivo. (A–C) Representative images of the basal turn of cochlear explants immunolabeled with MitoSOX (red) and myosin‐VIIa (green). Scale bar: 10 μm. (D) MitoSOX immunolabeling shows significant variations across treatment groups (*n* = 3). ***p < 0.001 versus control group; ^&&&^ *p* < 0.001 versus H_2_O_2_ group. (E–G) Immunolabeled 4‐HNE (red) and myosin‐VIIa (green) in OHCs of the middle turn of the cochlea in noise‐exposed C57BL/6J mice with or without DIDS supplementation and in the control group. Scale bar: 10 μm. (H) Quantification of 4‐HNE staining (*n* = 3). **p < 0.01 versus control group; *p < 0.05 versus 100 dB group.

Noise exposure induces the production of ROS and reactive nitrogen species in cochlear tissues, disrupting the balance between oxidants and antioxidants [30]. Oxidative stress triggers multiple cell death pathways in the cochlea, including apoptosis and necrosis in HCs and spiral ganglion neurons [31]. Thus, in this study, whether DIDS protects HCs by reducing H_2_O_2_‐induced oxidative stress was examined in cochlear explants. Mitochondrial ROS production was evaluated using the fluorescent probe MitoSOX [32]. The results showed that MitoSOX red fluorescence intensity was significantly higher in OHCs exposed to H_2_O_2_ than in the control group, but it was significantly reduced in explants treated with DIDS (Figure 5A–D).

Next, oxidative stress within the cochlear tissue was examined by assessing 4‐HNE expression, an indicator of oxidative stress [33]. Consistent with previous research [29, 34, 35], 4‐HNE levels in noise‐exposed cochlear OHCs were significantly elevated, whereas they were effectively reduced by DIDS (Figure 5E–H). These results suggest that DIDS can decrease oxidative stress both in vitro and in vivo.

### 3.5 DIDS Alleviates TNF‐α Expression in the Organ of Corti (OC)

**FIGURE 6:**
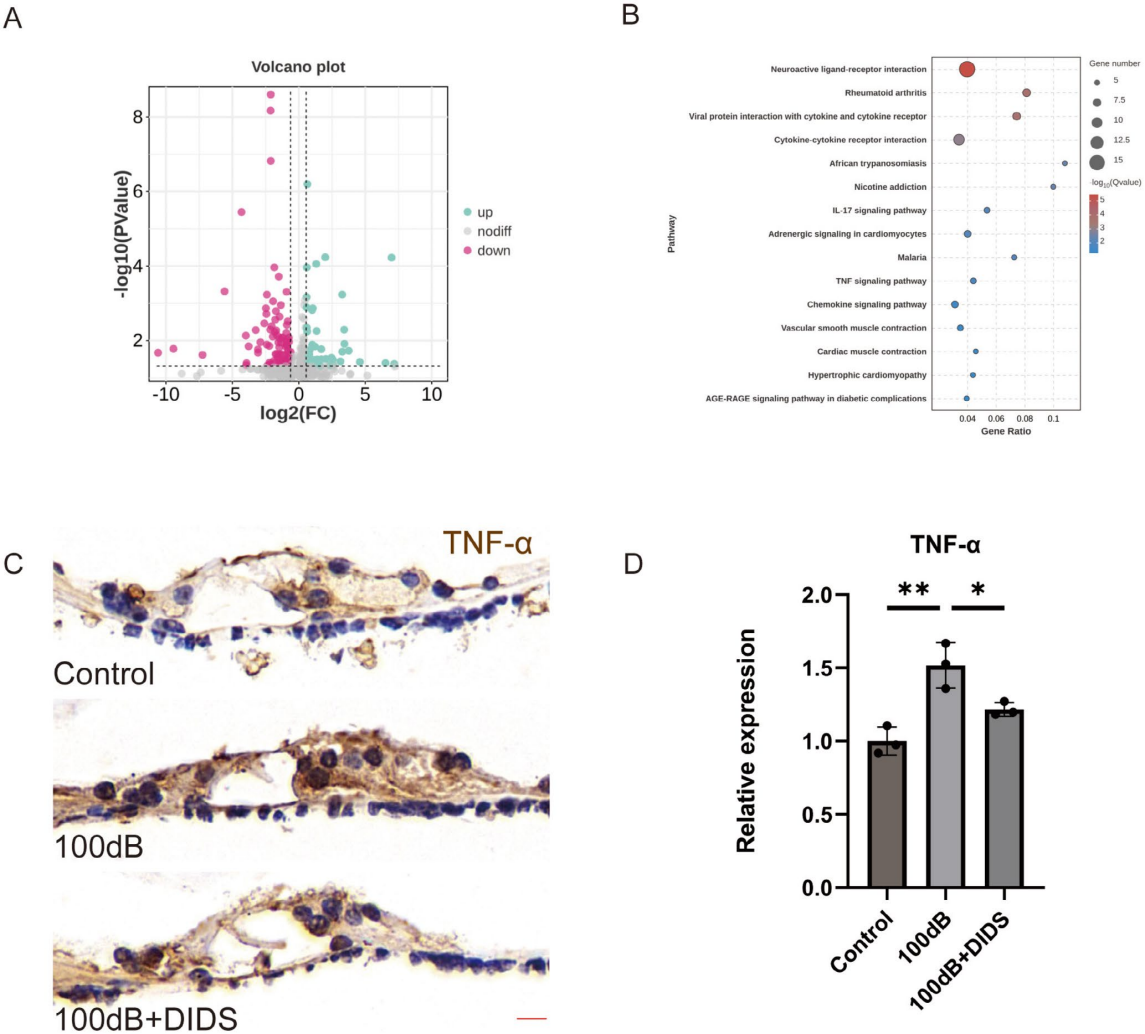
DIDS reduces TNF‐α expression in the organ of Corti (OC). (A) Volcano plot illustrating the results of RNA‐seq analysis, depicting the genes differentially expressed in the 100 dB + DIDS group versus the 100 dB group. (B) KEGG analysis shows the distribution of terms with statistically significant variances. (C) Representative immunohistochemical staining of TNF‐α in HCs. Scale bar: 5 μm. (D) Quantification of TNF‐α staining (*n* = 3). **p < 0.01 versus control group; *p < 0.05 versus 100 dB group.

It has been acknowledged that VDAC1 is associated with inflammation‐related pathways [36]. Whether DIDS alleviates the increase of inflammation in NIHL was examined using RNA sequencing (RNA‐seq), comparing transcriptomic changes in the cochlea of mice exposed to 100 dB noise, treated or not with DIDS. Compared with the 100 dB group, the 100 dB + DIDS group showed an increase in the expression of 46 genes and a decrease in the expression of 84 genes (Figure 6A). Figure 6B shows the top 15 enriched signaling pathways based on KEGG analysis. Among these pathways, the cytokine‐cytokine receptor interaction, chemokine signaling pathway, and TNF signaling pathway were considerably enriched. The pro‐inflammatory cytokine TNF‐α has been implicated in the pathogenesis of NIHL [37]. Elevated TNF‐α levels have been observed in the cochlea after noise exposure, and TNF‐α blockade can preserve hearing [38].

Consistent with the RNA‐seq results, immunohistochemical staining revealed a lower prevalence of diffuse TNF‐α staining in HCs of the 100 dB + DIDS group than in those of the 100 dB group (Figure 6C,D). These results show that DIDS reduces the noise‐induced elevation of pro‐inflammatory TNF‐α levels in vivo.

### 3.6 DIDS Protects Against Noise‐Induced Damage by Activating the PINK1/Parkin Pathway In Vivo

**FIGURE 7:**
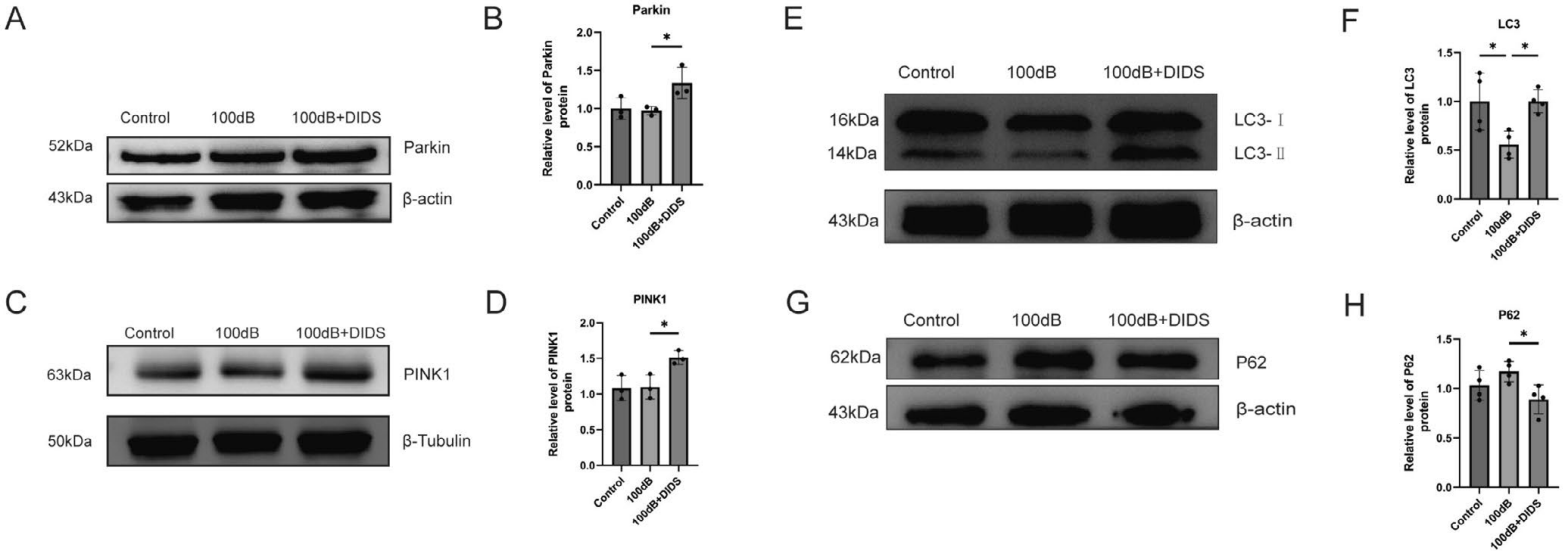
Elevated levels of mitophagy in noise‐exposed mouse cochlea treated with DIDS. (A, B) Western blot of Parkin expression in the cochlea. Expression levels were normalized against β‐Actin (*n* = 3). *p < 0.05 versus 100 dB group. (C, D) Western blot of PINK1 expression in the cochlea (*n* = 3). Expression levels were normalized against β‐tubulin. *p < 0.05 versus 100 dB group. (E, F) Western blot of LC3‐II expression in the cochlea. Expression levels were normalized against β‐Actin (*n* = 4). *p < 0.05 versus 100 dB group; *p < 0.05 versus control group. (G, H) Western blot of p62 expression in the cochlea. Expression levels were normalized against β‐Actin (*n* = 4). *p < 0.05 versus 100 dB group.

Recent studies have demonstrated that a decrease in VDAC1 levels induces mitophagy and autophagy in various cells and organs via the PINK1/Parkin pathway [20, 21, 39]. In this study, the levels of mitophagy‐associated proteins in the noise‐exposed mouse cochlea were assessed. Western blot assays showed elevated levels of PINK1 and Parkin in the cochlea following VDAC1 suppression by DIDS (Figure 7A–D). Inhibition of VDAC1 also led to a significant increase in LC3‐II levels, a key marker of autophagosomes, in the 100 dB + DIDS group compared with the 100 dB group (Figure 7E,F). Conversely, the expression of the autophagy substrate p62 was downregulated in the 100 dB + DIDS group compared with the 100 dB group (Figure 7G,H). The fluctuation in LC3‐II levels was inversely correlated with that of p62, indicating the maintenance of mitophagic flux.

### 3.7 Mitophagy Inhibition Exacerbates HC Damage and ROS in Cochlear Explants

**FIGURE 8:**
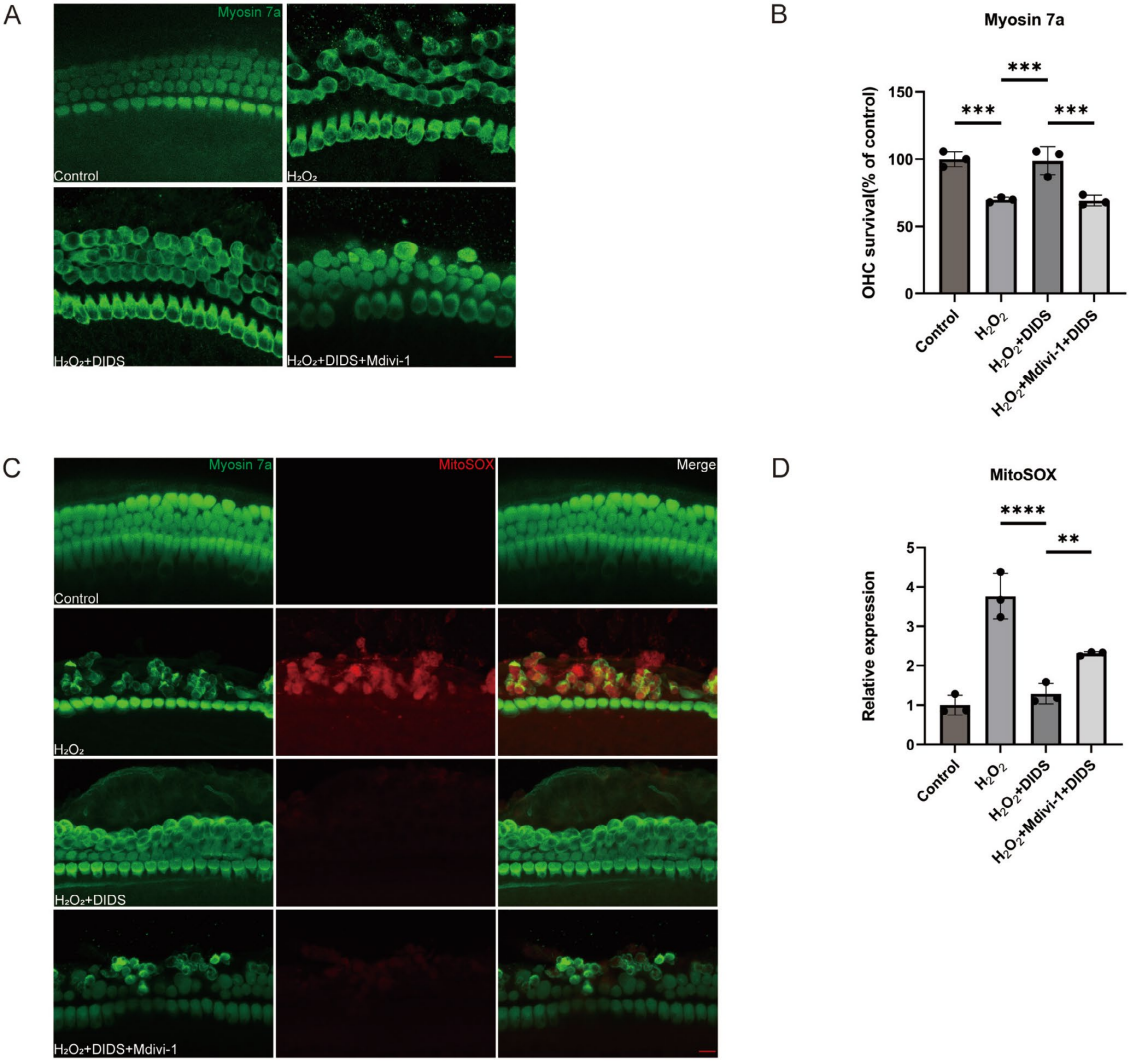
Mitophagy inhibition using Mdivi‐1 exacerbates HC damage and oxidative stress. (A) Representative images of myosin‐VIIa‐immunolabeled (green) basal turn cochlear explants in the different experimental groups. Scale bar: 10 μm. (B) Quantitative analysis of OHC survival in the different groups (*n* = 3). **p < 0.01 versus H_2_O_2_ group; *p < 0.01 versus H_2_O_2_ + DIDS group. (C) Representative images of the basal turn of cochlear explants immunolabeled with MitoSOX (red) and myosin‐VIIa (green). Scale bar: 10 μm. (D) MitoSOX immunolabeling shows significant variations across different groups (*n* = 3). ****p < 0.0001 versus H_2_O_2_ group; **p < 0.01 versus H_2_O_2_ + DIDS group.

Mitochondrial division inhibitor‐1 (Mdivi‐1), a suppressor of mitochondrial fission, is a valuable agent for assessing mitophagy. HC damage in cochlear explants was investigated by treating them for 24 h with 50 μmol/L Mdivi‐1, 0.5 mmol/L H_2_O_2_, and 20 μmol/L DIDS. The dose of Mdivi‐1 was based on the method used in a previous study [40]. Quantitative analysis showed a significant decrease in the survival of basal turn OHCs in the H_2_O_2_ group and the H_2_O_2_ + DIDS + Mdivi‐1 group compared with the H_2_O_2_ + DIDS group (Figure 8A,B). In addition, we verified the level of ROS by MitoSOX staining after using Mdivi‐1 (Figure 8C,D). The results showed that MitoSOX red fluorescence intensity was significantly higher in OHCs exposed to H_2_O_2_ + DIDS + Mdivi‐1 than in the H_2_O_2_ + DIDS group (Figure 8C,D). These results demonstrate the crucial role of mitophagy initiation in protecting OHCs against H_2_O_2_‐induced damage.

## 4 Discussion

NIHL is an important public health issue, causing 16% of disabling hearing loss worldwide [11], but an effective treatment remains unavailable. This study showed that VDAC1 inhibition protects against noise‐induced HC loss and synaptic ribbon damage in vivo and alleviates H_2_O_2_‐induced damage in vitro. Mechanistic analysis indicated that VDAC1 inhibition reduced noise‐induced inflammation and oxidative stress by activating mitophagy. To our knowledge, this is the first study to identify a role for VDAC1 in NIHL.

VDAC1, a protein located on the outer mitochondrial membrane, is involved in various cellular processes and has been implicated in several diseases [41]. In Alzheimer's disease (AD), VDAC1 is overexpressed in affected brain regions and is associated with neuronal cell death and mitochondrial dysfunction, key features of AD pathology [21]. Additionally, VDAC1 plays an important role in cardiovascular diseases, particularly in ischemia–reperfusion injury. VDAC1 overexpression in cardiomyocytes has been associated with increased susceptibility to cell death during ischemia–reperfusion events [41]. In type 2 diabetes, VDAC1 is overexpressed in pancreatic β‐cells and is linked to impaired cellular ATP generation, both of which may contribute to β‐cell dysfunction and insulin resistance [42]. A recent study revealed increased VDAC1 expression in auditory cells after cisplatin treatment [43]. Nonetheless, the role of VDAC1 in NIHL has not been extensively investigated.

In this study, single‐cell data showed that VDAC1 is widely expressed in different cell types of the cochlea. The high‐level expression of VDAC1 in cochlear HCs was confirmed by immunostaining. The increase in VDAC1 expression with increasing noise exposure indicated that overexpression of the protein is closely associated with NIHL. This hypothesis was tested in HEI‐OC1 cells and cochlear explants using in vitro H_2_O_2_ damage models and in vivo in a mouse model of NIHL. Both OHCs and the ribbon synapses of IHCs are vulnerable to insult in mammalian models of NIHL [44]. Our study showed that VDAC1 inhibition via DIDS protected against OHC and ribbon losses in mice and ameliorated the ABR shifts caused by noise. We also demonstrated the protective effects of DIDS in vitro through experiments using H_2_O_2_ in cochlear explants and HEI‐OC1 cells.

The pathogenesis of NIHL has been linked to mitochondrial dysfunction, where ROS play a pivotal role in damage to HCs and the auditory system [45]. Noise exposure induces mitochondrial damage and dysfunction in the cochlea, resulting in HC death and hearing loss [44]. After noise exposure, mitochondrial ROS production increases, leading to oxidative stress and auditory system damage [46]. Several studies have identified VDAC1 as a potential therapeutic target in mitochondrial dysfunction. For example, Ning et al. found that DIDS inhibition of VDAC1 protects neuronal mitochondria against silicon nanoparticle‐induced oxidative damage and dysfunction [47]. Experiments using SH‐SY5Y cells showed that VDAC1 inhibition improves mitochondrial function. Consistent with those reports, our results showed that 4‐HNE expression, a marker of oxidative stress, was significantly increased in HCs after noise exposure; this expression was inhibited by DIDS, indicating that VDAC1 inhibition protects against noise‐induced HC damage by reducing ROS production. In our model using H_2_O_2_ to simulate oxidative stress, VDAC1 inhibition decreased mitochondrial ROS production. Taken together, these findings suggest a role for VDAC1 in regulating mitochondrial function and subsequent ROS production, and thus in NIHL.

Inflammation, a complex biological response to harmful stimuli, has also been implicated in the pathogenesis of NIHL [38]. The proposed roles of VDAC1 in inflammation‐related disease pathogenesis include regulation of mitochondrial Ca^2+^ transport, lipid metabolism, and mitophagy [48]. VDAC1 is one of the most expressed and widely functional subtypes among the three discovered mammalian VDAC protein families [49]. A number of studies have demonstrated that the VDAC pore on the outer mitochondrial membrane allows mitochondrial DNA (mtDNA) generated after mitochondrial stress, to release into the cytoplasm [50, 51]. After being induced by mitochondrial ROS, cytoplasmic oxidized mtDNA could activate cGAS‐STING signaling, which triggered the activation of the NLRP3 inflammasome or the traditional NF‐κB signaling pathway and promoted the expression of pro‐inflammatory cytokine genes, including TNF‐α, IL‐6, and IL‐1β [36, 52, 53]. Modulation of overexpressed VDAC1 can mitigate brain pathology by inhibiting inflammation [54, 55]. However, whether inhibition of VDAC1 alleviates noise‐induced inflammation in the cochlea is not clear. Our RNA‐seq results showed that inflammation‐related pathways like cytokine‐cytokine receptor interaction, chemokine signaling pathway, and TNF signaling were primarily downregulated in the cochlea after DIDS treatment. Subsequent immunohistochemistry results showed an increase in TNF‐α levels in HCs after noise exposure, which was significantly decreased by DIDS. Similarly, Chen et al. found that SOX2 overexpression protected against NIHL by inhibiting inflammation, including levels of TNF‐α and interleukin‐6 [56]. Taken together, these results indicate that VDAC1 inhibition protects against NIHL by reducing TNF‐related inflammation.

Mitophagy, the selective degradation of dysfunctional mitochondria, is essential for maintaining mitochondrial quality control [57]. Several studies have shown that VDAC1 plays a pivotal role in regulating mitochondrial function and mitophagy [20, 21, 39, 58]. Another study revealed that Sert interrupts VDAC1 functions, reduces cellular ATP levels, activates AMPK, and inhibits MTOR, thereby inducing autophagy [20]. In the present study, the mechanism by which VDAC1 protects against NIHL was explored using DIDS, a pharmacological blocker of VDAC1 oligomerization. Our results revealed the involvement of the PINK1/Parkin pathway in DIDS‐mediated protection against NIHL in C57BL/6J mice. The subsequent activation of mitophagy mitigated the noise‐induced oxidative stress and inflammation in mouse HCs. An inverse correlation between p62, a marker of obstructed autophagic flux [59], and LC3‐II, a reliable marker of autophagosomes [60], was also identified, suggesting the preservation of mitophagic flux. Additionally, the inhibition of mitophagy via Mdivi‐1 administration nullified the protective effect of DIDS against H_2_O_2_‐induced injury and ROS in cochlear explants, providing further evidence that mitophagy mediates DIDS activity.

The role of the PINK1/Parkin pathway in the mitophagy‐mediated removal of damaged mitochondria has been extensively investigated in mammals [61]. In broad terms, mitochondria damaged by external stimuli lose their membrane potential, causing PINK1 to accumulate in the outer membrane of the impaired mitochondria, where it activates Parkin. Activated Parkin then facilitates the breakdown of damaged mitochondria by recruiting the autophagy adaptor and microtubule‐associated protein LC3, leading to the repair of dysfunctional mitochondria [61]. A recent study indicated that increased levels of VDAC1 monomers may provide additional anchor sites for the recruitment of PRKN‐mediated polyubiquitination, which then triggers mitophagy [39]. Therefore, the inhibition of VDAC1 in the cochlea may interfere with its oligomerization and promote PRKN/Parkin‐dependent mitophagy.

Given the widespread expression of VDAC1 in various tissues, systemic inhibition could lead to off‐target effects, impacting organs and systems not directly related to the intended therapeutic target. Inhibition strategies targeting VDAC1 may strike a delicate balance between therapeutic efficacy and minimizing off‐target effects. The broad functionality of VDAC1 raises concerns about unintended consequences of its systemic inhibition. To mitigate these risks, localized delivery methods should be prioritized. The inner ear is anatomically isolated within the bony labyrinth, in which the blood‐labyrinth barrier (BLB) restricts systemic drug penetration into inner ear tissues [62]. Local delivery methods, such as intratympanic injections or intracochlear administration, bypass the BLB but face other obstacles. For instance, diffusion across tissue barriers like the round window membrane and oval window remains inconsistent [62]. Nanoparticles can be engineered for targeted delivery to inner ear structures by enhancing drug stability and penetration through biological barriers [63]. Other novel strategies include microfluidic systems, natural multifunctional silk microcarriers, and cochlear prosthesis‐mediated delivery devices that allow for controlled administration directly into cochlear structures [64, 65, 66]. Therefore, combining these novel methods with VDAC1 inhibitors could be a potential strategy for NIHL in further clinical trials.

In conclusion, this study showed that VDAC1 is involved in NIHL and demonstrated the therapeutic potential of DIDS, a VDAC1 inhibitor, in its prevention and treatment by alleviating oxidative stress and inflammation in HCs. We found that VDAC1 inhibition activates mitophagy via the PINK1/Parkin pathway, thereby reducing noise‐induced HC and synaptic ribbon loss. Our findings provide a basis for further research concerning VDAC1 as a potential therapeutic target in the treatment of NIHL.

## Author Contributions

**Yuchen Jin:** data curation, formal analysis, writing – original draft, investigation. **Wenqi Dong:** methodology, visualization, validation. **Yumeng Jiang:** methodology, data curation. **Lingkang Dong:** writing – review and editing. **Zhuangzhuang Li:** project administration, supervision, writing – review and editing, data curation. **Dongzhen Yu:** resources, writing – review and editing, funding acquisition.

## Disclosure

G.E.O. data set: Gene expression data (GSE137299 profiling data) were downloaded from Gene Expression Omnibus (https://www.ncbi.nlm.nih.gov/geo/).

Animal studies: All animal experiments were conducted in accordance with the guidelines of the Institutional Animal Care and Use Committee of the Shanghai Sixth People's Hospital, affiliated with Shanghai Jiao Tong University School of Medicine.

## Conflicts of Interest

The authors declare no conflicts of interest.

## Data Availability

The datasets obtained and/or analyzed during the current study are available from the corresponding author upon reasonable request.
